# Sodium Hypochlorite and Diode Laser in Non-Surgical Treatment of Periodontitis: Clinical and Bacteriological Study with Real Time Polymerase Chain Reaction (PCR)

**DOI:** 10.3390/life12101637

**Published:** 2022-10-19

**Authors:** Marwan El Mobadder, Samir Nammour, Jacek Matys, Kinga Grzech-Leśniak

**Affiliations:** 1 Dental Surgery Department, Wroclaw Medical University, 50-425 Wroclaw, Poland; 2 Department of Dental Sciences, Faculty of Medicine, University of Liege, 4000 Liege, Belgium

**Keywords:** periodontal pathology, non-surgical treatment of periodontitis, laser, disinfection, oral cavity, pathogens

## Abstract

Increasing the disinfection during non-surgical treatment of periodontitis is primordial. This study assesses the effectiveness of sodium hypochlorite and a 980 nm diode laser in non-surgical treatment of periodontitis. Thirty sites of localized periodontitis with a probing pocket depth (PPD) of ≥ 6 mm were included. Fifteen underwent scaling root planing (SRP group) and 15 underwent SRP + 0.5% NaOCl and a 980 nm diode laser (study group). A biological molecular test and real time polymerase chain reaction (RT-PCR) were performed before (T0) and after intervention (T1). Total bacterial count and counts of Porphyromonas gingivalis, Treponema denticola, Tannerella forsythia, Prevotella intermedia, Peptostreptococcus micros, Fusobacterium nucleatum, Aggregatibacter actinomycetemcomitans, Eubacterium nodatum, Capnocytophaga gingivalis were assessed. Plaque index (PI), bleeding on probing (BOP), gingival recession (GR), PPD and clinical attachment loss (CAL) were evaluated at T0, and 3 and 6 months after. Study group showed a statistically significant reduction of TBC (5.66 × 10^8^ CFU/mL) compared to SRP (6.2 × 10^9^ CFU/mL). Both groups showed a statistically significant reduction of Treponema denticola, Tannerella forsythia, Prevotella intermedia, Peptostrep. (micromonas) micros and Fusobacterium nucleatum; however, a significant reduction of Eubacterium nodatum and Capnocytophaga gingivalis was observed in the study group. At T6, both groups had a statistically significant reduction of PI, BOP, GR, PD and CAL. The study group showed more GR compared to SRP and a significant reduction of PD (4.03 mm ± 0.49) compared to SRP (5.28 mm ± 0.67). This study reveals that NaOCl and a diode laser are effective as an adjunctive to the non-surgical treatment of periodontitis.

## 1. Introduction

Periodontitis is a chronic, biofilm-induced, multifactorial inflammatory disease of the periodontal tissues affecting the patients’ quality of life and their overall health [1,2,3]. Clinically, periodontitis is manifested by an apical migration of the junctional epithelium, a spreading of the bacterial biofilm along the root surface deep into the gingival sulcus, and a progressive destruction of the periodontal tissue, specifically the periodontal ligaments and the alveolar bone [4,5]. Studies suggest that the progression of periodontitis depends fundamentally on the shift of the balanced symbiotic microbiota [6] that is found physiologically in the sulcus to a dysbiotic one dominated by Gram-negative anaerobic microbiota in which *Porphyromonas gingivalis* is described as being a keystone pathogen [7]. In fact, it is the interaction between this microbiota and the host response that eventually leads to a complex inflammatory exchange that can either remain in the stage of gingivitis or for some known and unknown causes progress to periodontitis [6,7]. In other words, the dysbiotic biofilm alone is not sufficient to induce periodontitis and it is the host’s inflammatory response to this microbial challenge that can ultimately cause the destruction of the periodontium [6,7]. 

More than 700 bacterial species are found in subgingival biofilm; however, not all of them are responsible for the initiation and/or progression of periodontitis [8,9,10]. For instance, it is now well established that *Porphyromonas gingivalis*, *Treponema denticola*, and *Tannerella forsythia* are implicated significantly in the development and progression of periodontitis. In addition to the already mentioned bacteria, *Eikenella corrodens*, *Prevotella* species, *Fusobacterium nucleatum*, *Peptostreptococcus micros* and *Campylobacter rectus* are also found abundantly in deep periodontal pockets and are implicated as well as possible periodonto-pathogens [11].

Based on the European Federation of Periodontology’s clinical practice guideline, non-surgical treatment consisting of mechanical debridement with ultrasonic and manual instrumentation remains the gold standard treatment of stage I-III periodontitis [12,13]. The aim is to eradicate, as much as possible, the supra- and subgingival biofilm and calculus from the colonized root surfaces, to reduce the PPD and to re-establish the clinical attachment level [13,14]. However, the non-surgical treatment might present several limitations depending on the presence of deep periodontal pockets, the accessibility of the manual and ultrasonic instruments in these deep pockets and the presence of morphological challenges such as fissures, concavities and furcation involvement [15,16,17]. Scaling and root planing (SRP) alone offer mechanical debridement of calculus and biofilm by direct contact of hard and soft tissues which can be considered a limitation in deep pockets and/or in some challenging to reach anatomical areas. To compensate for the limitations of the non-surgical treatment, additional approaches were suggested in the literature such as the use of antibiotics [18], disinfection solutions [19], antimicrobial peptides [20], probiotics [21,22], laser irradiation [19,23,24] and photodynamic therapy [25,26]. In this context, sodium hypochlorite (NaOCl) [27] and a diode laser [28] are proposed as an additional approach for SRP in the non-surgical treatment of periodontitis [27,28]. NaOCl presents a broad antimicrobial activity with a high dissolving effect on necrotic tissue and fast bactericidal action that has been known for many years [29]. Laser irradiation, if used adequately, might significantly increase the disinfection of deep periodontal pockets as its energy penetrates in depth, increasing the disinfection of the periodontium [19,23,30]. Moreover, some studies of root canal therapy suggest that the combination of a diode laser and NaOCl can increase the disinfection of the root canal system by causing a direct heating of NaOCl leading to an increase in its activity [31]. Coupling a 980 nm diode laser and 0.5% NaOCl might result in both desired advantages: a better disinfection by increasing the temperature and by direct disinfection from the energy of the diode laser.

Hence, the aim of this study was to assess the disinfection potential of combining a 980 nm diode laser and 0.5% NaOCl in non-surgical treatment of periodontitis. A biological molecular test with real-time polymerase chain reaction (PCR) was performed before and immediately after treatment for both groups. In addition, plaque index (PI), bleeding on probing (BOP), gingival recession (GR), PPD and clinical attachment loss (CAL) were evaluated before intervention, at 3 months and at 6 months after intervention for both groups. The null hypothesis was that there is no difference in the disinfection potential nor in the clinical periodontal parameters (PI, BOP, GR, PD and CAL) between SRP alone vs. SRP + 0.5% NaOCl + diode laser irradiation.

## 2. Materials and Methods

### 2.1. Study Design and Registration

Standard parameters including a significance level of 0.05, d value of d = 0.82, 95% confidence interval and 70% power of the study were used to calculate a sample size equal to 15 for each group using G×Power software (Kiel University, Kiel, Germany). Thirty patients’ sites of localized periodontitis with a probing pocket depth (PPD) of ≥ 6 mm were included. After obtaining informed consent, the included patients *(n =* 30*)* were randomly assigned to one of two groups using a computer-generated number sequence. This random selection was concealed until group assignment. Patients, operators, and investigators were not blinded to the assignment. This study was made according to the Declaration of Helsinki. All patients were recruited between November 2020 and May 2021. All patients signed a written informed consent form before their enrollment. This study was approved by the Bioethics Committee at the Medical University of Wroclaw, appointed by the order of the Rector of the Medical University of Wroclaw No. 278/XVI on 21 December 2020. The study included a control group consisting of scaling root planing intervention only (SRP only) and a study group consisting of NaOCl irrigation and 980 nm diode laser irradiation immediately after SRP (SRP + NaOCl + laser).

#### 2.1.1. SRP Group 

In this group, oral hygiene instructions were provided and the motivation for adequate oral hygiene was presented. Conventional scaling root planing (SRP) with ultrasonic and manual instrumentation was made. This group consisted of 15 patients (*n* = 15) and was considered the control group. The detailed protocol created for this group is presented in Section 2.3.1.

#### 2.1.2. SRP + NaOCl + Laser Group 

In addition to what was created for the SRP group, 0.5% NaOCl was introduced in the periodontal pocket for 30 s and then activated with the 980 nm diode laser for 60 s. This group consisted of 15 patients (*n* = 15) and was considered the study group. Details on the protocol created for this group are found in Section 2.3.2.

### 2.2. Inclusion and Exclusion Criteria

#### 2.2.1. Inclusion Criteria

- Localized periodontal pocket with a probing depth ≥ 6 mm and a clinical attachment loss of > 3 mm (periodontitis stage III or IV; classification of the European Federation of Periodontology and American Academy of Periodontology);
- Patients who signed the written informed consent and is willing to cooperate;
- Bleeding on probing in more than 30% of the sites.

#### 2.2.2. Exclusion Criteria

- Medical history likely to affect periodontal status and/or to compromise treatment outcomes;
- Patients who received antibiotic and/or anti-inflammatory treatments the last 6 months;
- Patients who received immunosuppressors treatments the last 6 months;
- Patients who received any periodontal therapy for the last 6 months;
- Pregnant or lactating women;
- Smokers smoking more than 10 cigarettes per day.

### 2.3. Treatment Protocol

#### 2.3.1. Conventional Treatment for All Groups (*n* = 30)

Oral hygiene instructions were provided to all included patients *(n =* 30*)* and motivation for adequate oral care was presented. After that, mechanical debridement was performed with ultrasonic and manual instrumentations. Professional scaling and root planing (SRP) were performed with an ultrasonic piezoelectric scaler (Piezosteril 6, Castellini, Cazzago San Martino, Italy) for all teeth and manual instrumentation with curettes Gracey (Universal and Gracey curettes) was performed only for periodontal pockets that were deeper than 5 mm. After mechanical debridement, a 0.12% Chlorhexidine solution (Eludril pro, mouthwash, Pierre Fabre Oral Care, Paris, France) was applied to the areas with periodontal pockets that were deeper than 5 mm. After the mechanical debridement, interdental brushes with specific shapes and sizes were prescribed to be used. After treatment, education and motivation were provided for proper in-home oral hygiene and a 0.12% chlorhexidine mouthwash was prescribed for 7 days for all the patients. 

#### 2.3.2. Group SRP + NaOCl + 980 nm Laser (*n* = 15)

This protocol was applied only for the SRP+ NaOCl + laser group and immediately after the end of the conventional treatment that was performed for all patients. The following steps were taken for this group:

- Irrigation of periodontal pocket with NaOCl (0.5%) for 30 s using a syringe with a needle of 0.3 × 25 mm. During irrigation, aspiration was performed to ensure no NaOCl will pass outside the pocket; hence, avoiding its contact with the oral tissue to prevent side effects such as the unpleasant sensation on the patient;
- After 30 s of irrigation, the fiber of the diode laser 980 nm (Smart M, Lasotronix, Warsaw, Poland) was inserted into the pocket until 1 mm above the bottom of the pocket;
- Irradiation was performed within an inward and outward movement parallel to the longitudinal axe of the tooth in contact mode with the inner pocket epithelium, a frequency of 10 kHz, a pulse duration of 10 µsec, a pick power of 10 W, average power of 1 W, and a fiber diameter of 320 µm for an average time of 60 seconds of total irradiation;
- The same procedure was repeated three times.

### 2.4. Bacteriological Study

Thirty microbiological samples were collected from the periodontal pockets of both groups before intervention (T0) and immediately after intervention (T1). Microbiological sampling consisted of total bacteria count (TBC) and the count of *Porphyromonas gingivalis*, *Treponema denticola*, *Tannerella forsythia*, *Prevotella intermedia*, *Peptostreptococcus micros*, *Fusobacterium nucleatum*, *Aggregatibacter actinomycetemcomitans*, *Eubacterium nodatum* and *Capnocytophaga gingivalis.* The total bacterial count (TBC) is the quantitative estimate of the number of the microorganisms present in the samples; its measurement is represented by the number of colony-forming bacterial units (CFU) per mL. The bacteriological study for TBC and the count of each of the periodonto-pathogens was conducted by removing the supragingival plaque with sterile cotton pellets without inducing bleeding then inserting sterile paper points into the periodontal pockets in accordance with the procedure recommended by the manufacturer (Pet Plus, MIP Pharma, Germany). The paper points were then put into a transport Eppendorf tube specifically made for the PET plus kit, and were shipped to MIP Pharma laboratory in Germany, where samples processing was performed. The exact protocol for the bacteriological study is confidential for the company; however, PET Plus is a CE certified medical device, manufactured by MIP Pharma in Germany. At the laboratory, the samples were analyzed as a cumulated sample using the real-time polymerase chain reaction (PCR) technique. Free strand sections of DNA were obtained from lysed bacterial cells and were subsequently subjected to amplification and hybridization with fluorescence-stained starters characteristics of particular periodonto-pathogens. Quantitative analysis was performed with a reader that measures the intensity of fluorescence compared to that in the reference specimens. According to the manufacturer, the threshold determination for all subjects’ bacterial pathogens was approximately 10^3^ bacteria. The threshold determination adopted in this research was approximately 10^4^ bacteria. The PCR-based test utilizes the fast duplication of selected DNA/RNA strand sections as products and allows quantitative analysis of the copied products within the study’s samples. 

### 2.5. Clinical Periodontal Parameters

A single calibrated examiner (M.E.M) recorded all clinical measurements for both groups before intervention (T0), 3 months post-intervention (T3) and 6 months post-intervention (T6). The before-intervention data were collected on the same day of treatment. Plaque index (PI), bleeding on probing (BOP), gingival recession (GR), probing pocket depth (PPD) and clinical attachment loss (CAL) were evaluated. PI (Silness-Löe 1964) was calculated by giving a score from 0 to 3 on four surfaces of teeth #16, #12, #24, #36, #32, and #44. The scores from the four areas of the tooth were added and divided by four. BOP, GR, PD and CAL were measured using a periodontal probe (PCP UNC 15, HuFriedy, Chicago, USA). BOP was evaluated on six sites for each tooth and the scores were expressed as a percentage of the number of gingival units that bleed related to the total number of units. GR and PD were assessed on six sites per treated tooth (mesio-buccal, buccal, distobuccal, mesiopalatal/lingual, palatal/lingual, and disto-palatal/lingual) and were measured in mm. Clinical attachment loss was calculated by adding the probing depth to the gingival margin level if a recession is present and by subtracting the gingival margin level from the probing depth when the gingival margin was coronal to the CEJ. All parameters were measured and recorded before intervention (T0), at 3 months of the intervention (T3) and six months of intervention (T6).

### 2.6. Statistical Analysis

For the statistical analysis, Sigma five^®^ software was used (GraphPad Prism 5, San Diego, CA, USA). Statistical significance was considered when the *p* value was <0.001, which is a very high significance. Mean and standard deviation (Std) were calculated for each group before intervention (T0) and immediately after intervention (T1) for mean values and standard deviations (SD) of PI, BOP, GR, PD and CAL. The mean value of the total bacterial count (TBC) was obtained for each group at T0 and T1 and the mean and standard deviation of the bacterial count for each included bacterial were also measured for each group at T0 and T1. Smirnov and Kolmogorov tests were utilized to assess the normality tests. One-way ANOVA coupled with a Newman–Keuls multiple comparison test (post hoc test) was used to assess the presence or absence of a statistically significant difference within groups at T0 and T1 and between groups. Standard parameters including a significance level of 0.05, d value d = 0.82, 95% confidence interval and 70% power of the study were used to calculate a sample size equal to 15 for each group using the G×Power software (Kiel University, Kiel, Germany).

## 3. Results

### 3.1. Results of the Total Bacterial Count

Before intervention, there was no statistically significant difference between the total bacterial count (TBC) of both groups: 1.4698 × 10^11^ CFU/mL and 1.26939 × 10^11^ CFU/mL for the SRP group and the SRP + NaOCl + laser group, respectively. After intervention, there was a reduction in both groups; yet, the reduction was significantly higher for the SRP + NaOCl + laser when compared to the SRP group: 6.2 × 10^9^ CFU/mL and 3.723 × 10^8^ CFU/mL for the SRP group and the SRP + NaOCl + diode laser group at T1. Therefore, the suggested protocol resulted in an overall significantly higher disinfection when compared to conventional mechanical debridement (Table 1). 

### 3.2. PCR Test Results

#### 3.2.1. SRP Group

Scaling and root planing resulted in a statistically significant reduction of *Treponema denticola*: 319,110 count at T0 vs. 5560 count at T1, *Tannerella forsythia* (13,036 count at T0 vs. 952 count at T1), *Prevotella intermedia* (413,690 count at T0 vs. 1413 count at T1) and *Fusobacterium nucleatum* (21,130 count at T0 vs. 0 count at T1). Although a reduction was observed for all other included pathogens, this reduction was not statistically significant between T0 and T1 for *Aggregatibacter actinomycetemcomitans (*29.6 count at T0 vs. 0 count at T1), *Porphyromonas gingivalis* (27,310 count at T0 vs. 2530 count at T1), *Eubacterium nodatum* (2820 at T0 vs. 1000 at T1) and *Capnocytophaga gingivalis* (50,990 at T0 vs. 8680 at T1) (Table 2).

#### 3.2.2. SRP + NaOCl + Laser Group

For the study group, statistically significant reductions of *Treponema denticola* (234,242 at T0 vs. 2260 at T1), *Tannerella forsythia* (148,485 at T0 vs. 151 at T1), *Prevotella intermedia*, *Peptostrep. (micromonas) micros* (1684 at T0 vs. 0 at T1), *Fusobacterium nucleatum* (19,764 at T0 vs. 0 at T1), *Eubacterium nodatum* (3095 at T0 vs. 0 at T1) and *Capnocytophaga gingivalis* (68,699 at T0 vs. 859 at T1) were observed when T1 was compared to T0 (Table 2).

### 3.3. Results of the Periodontal Parameters for Both Groups and at Different Times of Follow-Up

#### 3.3.1. Plaque Index

A significant reduction of plaque index was obtained at 3 and 6 months of follow-up compared to T0 for both groups. Within groups, there was no statistically significant difference in PI values at all times of follow-up (Table 3).

#### 3.3.2. Bleeding on Probing

Both groups resulted in a significant reduction of BOP; however, there was a statistically significant difference at six months of follow-up between the SRP + NaOCl + laser group (14.32% ± 9.07) and SRP only (20.70% ± 9.44) (Table 3).

#### 3.3.3. Gingival Recession

The suggested protocol (NaOCl and laser) resulted in a significant GR (0.81 ± 0.30, 1.72 ± 0.51, 1.73 ± 0.54 for T0, T3 and T6 respectively) while SRP only did not result in a gingival recession (no significant difference between T0, T3 and T6 values) (Table 3). Within groups, a significant difference in GR was observed in the SRP + NaOCl + laser group compared to SRP only.

#### 3.3.4. Probing Pocket Depth

Mean values of probing pocket depth significantly decreased in both groups from 7.92 ± 0.93 mm at T0 to 5.28 ± 0.67 mm at six months follow-up for SRP only and from 7.99 ± 0.83 mm to 4.03 ± 0.49 mm at six months of follow-up. Additionally, within groups, a significant closure of the periodontal pocket was obtained for the SRP + NaOCl + laser group compared to only SRP after 6 months.

#### 3.3.5. Clinical Attachment Level

Both treatments resulted in a significant reduction of the clinical attachment loss (CAL). Values of CAL obtained for SRP group were 8.76 ± 1.32 mm at T0 and 6.32 ± 1.3 at 6 months. For the SRP + NaOCl + laser group, values were 8.8 ± 1.13 at T0 vs. 5.76 ± 1.03 at 6 months. Within groups, a significant difference can be noted between SRP alone and SRP + NaOCl + laser in which a greater decrease in the clinical attachment loss can be observed when the suggested protocol was used (Table 3). 

## 4. Discussion

Based on the clinical periodontal parameters, this study showed that the use of 0.5% sodium hypochlorite combined with 980 nm diode laser irradiation results in a statistically significant reduction in the probing pocket depth (PPD) and an improvement in the clinical attachment level of deep periodontal pockets (≥6 mm). The obtained PPD values at six months were statistically significantly lower than those obtained in SRP only with 5.28 ± 0.67 and 4.03 ± 0.49 respectively for the PPD of the study and control groups. However, a significant gingival recession was observed when the suggested protocol was used (Table 3). As for the microbiological study, the suggested protocol showed an overall significant reduction of the total bacterial count. This reduction in the TBC was significantly greater than that obtained with SRP alone (5.6 × 10^8^ for study group vs. 6.2 × 10^9^ for SRP only after treatment).

In fact, before intervention, there was no statistically significant difference in the TBC of both groups: 1.4698 × 10^11^ CFU/mL and 1.26939 × 10^11^ CFU/mL for the SRP group and the SRP + NaOCl + laser group, respectively. After intervention, a significantly higher reduction was obtained for SRP + NaOCl + laser (3.723 × 10^8^ CFU/mL) when compared to the SRP group (6.2 × 10^9^ CFU/mL). The PCR test revealed a significant reduction of *Treponema denticola*, *Tannerella forsythia*, *Prevotella intermedia*, *Peptostrep. (micromonas) micros* and *Fusobacterium nucleatum* counts was obtained when scaling root planing was made while SRP + NaOCl + laser resulted in a significant reduction of the same mentioned bacteria in addition to *Eubacterium nodatum* and *Capnocytophaga gingivalis* counts. Hence, the findings of the study propose that 0.5% NaOCl and irradiation with the 980 nm diode laser increases the overall disinfection of deep periodontal pockets (≥ 6 mm) during non-surgical treatment which is manifested by a significant reduction of TBC and a significant reduction of seven of the nine periodonto-pathogens. On the other hand, the clinical periodontal parameters additionally showed that the suggested protocol resulted in a significant reduction of the PPD after treatment and within six months of follow-up (4.03 ± 0.49 mm) compared to SRP alone (5.28 ± 0.67 mm). However, it is important to note that the suggested protocol resulted in a significant gingival recession, which can be considered a shortcoming of the protocol. This important GR might have been due to the important resolution of the inflammatory process or due to the heating that the irradiation with the laser provokes. As for the plaque index and the bleeding on probing, both SRP and SRP + NaOCl + laser groups showed values after treatment and within six months of follow-up that indicates an important resolution of the inflammatory process and satisfactory plaque control. 

The aim of using additional approaches such as NaOCl and a laser is to improve the clinical outcome of the non-surgical treatment of periodontitis by improving the disinfection; thus, limiting the need for a surgical intervention. *Porphyromonas gingivalis* was suggested to be a keystone pathogen in periodontitis due to its remarkable immune subversion activities and its crucial role in periodontal dysbiosis [11]. The aim of this protocol was to add to the mechanical debridement, a chemical (NaOCl) disinfection and energy (light) that can penetrate beyond the limit of the mechanical debridement obtained with ultrasonic instrumentation. For instance, irradiation with a diode laser generates thermal and photo-disruptive energy leading to a sublethal damage of the bacteria present in contact with and near the site of irradiation [32]. The heat and photo-disruptive energy can reach deep areas that the conventional mechanical debridement cannot [32,33,34]. For instance, this might have increased the disinfection of the epithelial junction, the connective tissue and the periodontal ligaments [19,32,33,34]. In addition, the 980 nm wavelength is in the near infrared light that was shown to be able to directly kill pigmented bacteria that contain protoporphyrin IX such as *Aggregatibacter actinomycetemcomitans* [35]. Moreover, this deep penetration of the energy can result in a deep detoxification and decontamination of the periodontal pocket with the ability to eradicate periodontal pathogens trapped within the gingival epithelial cells. On the other hand, sodium hypochlorite is very well known in its strong organic dissolution and its potent antimicrobial activity [29,31]. Its effectiveness comes from “chlorine”, a substance formed from the contact of hypochlorous acid with organic tissue [29]. NaOCl presents a strong ability to inhibit bacterial enzymes leading, thus, to an irreversible oxidation of Sulphydryl group that are essential for bacterial enzymes [29]. NaOCl was used in the deep pockets in an attempt to detoxify the root surfaces and to soften the calculus, thus facilitating its removal by means of root planing [36,37,38,39]. Another advantage of combining NaOCl and a diode laser might have been the heating of NaOCl when laser irradiation is activated [36,37,38,39]. In fact, the thermal effect on NaOCl led to the formation of a chemical reaction that results in an excessive release of oxygen [40,41]. This catalytic-photothermal antibacterial strategy of combining NaOCl and a 980 nm diode laser had the aim of accelerating the recovery of infected periodontal wound by increasing the disinfection. As for the safety of our suggested protocol, both the use of the diode laser within our parameters and the use of a 0.5% concentration of NaOCl were demonstrated to be safe for the periodontal tissues in numerous studies [42,43]. In addition, since deep pockets (>5 mm) with periodontitis were included, the bacteria involved were mainly Gram-negative, found in the periodontal pocket. These Gram-negative bacteria possess tough cell walls made of highly cross-linked murein, making them more resistant to laser irradiation alone [36,37,38,39,40,41,42]. Therefore, the combination of both laser and NaOCl might have overpassed the walls of the anaerobic bacteria making them less resistant and more vulnerable to the disinfection process. It is reasonable to expect better disinfection potential when two different approaches are carried out in the same area because each agent (laser or NaOCl) will target different and specific pathogens based on its characteristics and interaction and thus their combination will result in a broader disinfection. 

The PCR test used in our study revealed an important finding. The suggested protocol was more effective on the overall bacterial flora compared to SRP alone but this significant difference was not always obtained for the nine periodonto-pathogen (based on the PCR results). This important finding suggests that the present protocol presents an overall bactericidal effect on all the present pathogens in the periodontal pocket and not only on the red complex. Hence, it must have been that the use of sodium hypochlorite and a 980 nm diode laser significantly eradicated bacteria from the green and orange complex and orange-associated complex. However, the suggested protocol did not have significant bactericidal activity on *Porphyromonas gingivalis* when the count after intervention was compared to SRP alone (29995 for before vs. 5.66E+8 for after intervention). In this context, we suggest further studies to be performed with a larger number of patients aiming to assess more adequately the effect of our suggested protocol on the count of *Porphyromonas gingivalis*. *Porphyromonas gingivalis* as “the keystone pathogen” might be resistant, suggesting that the treatment will not ultimately lead to a complete resolution of the periodontitis; this is why a clinical long-term investigation with a higher number of participants is recommended.

In the literature, there is a relatively large number of studies related to the use of different concentrations and forms of sodium hypochlorite and/or different protocols and parameters of laser irradiation in addition to mechanical debridement in non-surgical periodontal treatment. For instance, Grzech-Leśniak showed that the combination of an Nd: YAG laser and 0.5% NaOCl results in a significant reduction of *Porphyromonas gingivalis*, *Fusobacterium nucleatum* and *Streptococcus gordonii’s* viability when compared to SRP alone [42]. The findings were not in accordance with our study in which it was found that no significant reduction of *Porphyromonas gingivalis* was found. This might be attributed to the fact that Nd:YAP and the diode laser present different tissue interactions and are absorbed differently by water, hemoglobin and pigmentation. Moreover, an in vitro study has shown that a formulation consisting of NaOCl 0.95% and amino acids (glutamic acid, leucine, lysine) presents a strong antimicrobial effect, in particular against Gram-negative species associated with periodontitis [44]. Subsequent findings from in-vitro studies [44,45] have shown that the application of amino acid-buffered hypochlorite solutions can have a positive effect on the survival, attachment and spreading of periodontal ligament cells onto root surfaces [45]. In this context, Jurczyk et al. [46] showed in an in-vitro study that NaOCl gels are strong antimicrobial agents in particularly against Gram-negative species associated with periodontitis. Another study by Nibali et al. [47] reported that a mean value of 2.93 mm of bone augmentation was observed radiographically at sites associated with intrabony defects treated by means of minimally invasive nonsurgical therapy combined with NaOCl irrigation. The most widely used irrigation solution in periodontal therapy remains chlorhexidine; however, other irrigation solutions were proposed and investigated such as hydrogen peroxide (generally 3%), povidone-iodine, fluoride mouthwash and others. In this context, El Mobadder et al. [19] showed in a retrospective study, including 128 sites of localized periodontitis, that the use of 3% of hydron peroxide coupled with irradiation with a 980 nm diode laser can provide a significant reduction in the TBC of periodontal pockets that are greater than 5 mm. However, El Mobadder et al. [19] did not assess the bacterial count of the specific periodonto-pathogens; thus, their suggested protocol might not be as effective on the red complex as it was on the total bacteria present in the deep periodontal pockets. On the other hand, Grzech-Lesniak showed in a randomized clinical and microbiological study that multiple applications of aPDT using toluidine blue 0.1% as a photosensitizer can be an effective additional approach to the SRP during maintenance sessions for patients with periodontitis [48]. Bansal et al. [49] found that the use of a laser and chlorhexidine chip assures a significant reduction in TBC when compared to SRP alone. However, the follow-up after treatment was carried out for only 4 weeks [49]. G. Caccianiga et al. showed in a microbiological study that using a laser with hydrogen peroxide has a significant bactericidal effect on *Prevotella intermedia*, *Peptostreptococcus micros* and *Fusobacterium nucleatum* and that the best results can be obtained if both hydrogen peroxide and a diode laser are combined on these three periodonto-pathogens [50].

In this study, the effectiveness in terms of disinfection potential was measured accurately since not only was TBC calculated before and immediately after treatment but also the periodontal pathogens linked to the development and progression of periodontitis were specifically measured using the real time PCR test. Based on the literature, this is the first study to clinically assess the disinfection potential of NaOCl activated with a 980 nm diode laser using the real time polymerase chain test for the management of deep periodontal pockets (≥6 mm).The bacteriological and clinical results obtained are promising; the protocol can be considered a non-invasive approach and might help clinicians to avoid surgical intervention. Hence, 0.5% NaOCl coupled with a 980 nm diode laser can be used clinically as an adjunctive approach for the gold standard mechanical debridement in a pocket with a depth of more than 5 mm. This adjunctive protocol might help with obtaining a resolution of the inflammatory process leading ultimately to the resolution of periodontitis. In further studies, a larger sample size with a longer follow-up period (>6 months) can better reflect the impact of the suggested protocol on the overall resolution periodontitis. Hence, due to the promising and significant bactericidal effect obtained, we invite further long-term randomized clinical trials with large sample size studies to be performed in order to confirm the effectiveness of the current suggested protocol. Moreover, different NaOCl concentrations and irradiation parameters might result in different clinical outcomes.

## 5. Conclusions

At six months of follow-up, the suggested protocol resulted in a significant reduction of PPD, a significant improvement in clinical attachment level but a significant increase in GR compared to scaling root planing alone. Moreover, the microbiological study showed that the suggested protocol resulted in a significant reduction of TBC compared to SRP and a significant reduction of the counts of *Treponema denticola*, *Tannerella forsythia*, *Prevotella intermedia*, *Peptostrep. (micromonas) micros*, *Fusobacterium nucleatum*, *Eubacterium nodatum* and *Capnocytophaga gingivalis*.

## Figures and Tables

**Table 1:** Mean and standard deviation of total bacterial count (TBC) for both groups.

	Before Intervention		Immediately after Intervention	
	Mean Value	Std	Mean Value	Std
**SRP**	1.47 × 10^11 A^	1.29 × 10^11^	6.2 × 10^9 B^	6.1 × 10^9^
**SRP + NaOCl + laser**	1.89 × 10^11 A^	1.33 × 10^11^	5.6 × 10^8 C^	4.9 × 10^8^

Identical letters of exponent (^A, A^) or (^B, B^) or (^C, C^) etc. indicates the absence of a statistically significant difference, while the difference in exponent letters (^A, B^) or (^B, C^) or (^A, C^) etc. indicates a statistically significant difference. *p*-value < 0.0001, all values are in CFU/mL. Mean = mean value of the total bacterial count obtained for each group; Std = standard deviation of the total bacterial count obtained in each group.

**Table 2:** Mean value and standard deviation of the biological molecular test and real time polymerase chain reaction test before and immediately after treatment in both groups.

	SRP Group				SRP + NaOCl + Laser			
	before Intervention		Immediately after Intervention		before Intervention		Immediately after Intervention	
	Mean	Std	Mean	Std	Mean	Std	Mean	Std
Aggregatibacter actinomycetemcomitans	**29.6** ^a^	18.6	**0**	0	**31.42** ^a^	21.42	**0** ^a^	0
Porphyromonas gingivalis	**27310** ^a^	19580	**2530** ^a^	1992	**29995** ^a^	17575	**2260** ^a^	1832
Treponema denticola	**319110** ^a^	389880	**5560** ^b^	3560	**234242** ^a^	195329	**1376** ^b^	932
Tannerella forsythia	**13036** ^a^	9989	**952** ^c^	422	**148485** ^b^	9389	**151** ^c^	99
Prevotella intermedia	**413690** ^a^	391100	**1413** ^b^	1413	**393414** ^a^	393414	**0** ^c^	0
Peptostrep. (micromonas) micros	**11553** ^a^	9923	**2102** ^b^	1834	**1684** ^b^	984	**0** ^c^	0
Fusobacterium nucleatum	**21130** ^a^	18930	**0** ^b^	0	**19764** ^a^	13949	**0** ^b^	0
Eubacterium nodatum	**2820** ^a^	1850	**1000** ^b^	1000	**3095** ^a^	2994	**0** ^b^	0
Capnocytophaga gingivalis	**50990** ^a^	42553	**8680** ^b^	7980	**68699** ^a^	59363	**859** ^c^	647

*p*-value < 0.0001. Identical letters of exponent (^a^^,^^a^) or (^b, b^) or (^c, c^) etc., indicates the absence of a statistically significant difference, while the difference in exponent letters indicates a statistically significant difference (^a–c^). Mean = mean value of the bacterial count obtained for each group; std = the standard deviation of the bacterial count obtained for each group. T0 = before treatment; T1 = immediately after treatment.

**Table 3:** Results of the clinical periodontal parameters for SRP group and SRP+ NaOCl + laser at different times of follow-up.

Variables	SRP Group	SRP + NaOCl + Laser
**Plaque index**		
T0	1.77 ± 0.74 ^a^	1.87 ± 0.77 ^a^
T3	1.30 ± 0.54 ^b^	1.11 ± 0.43 ^b^
T6	1.37 ± 0.48 ^b^	1.32 ± 0.45 ^b^
**Bleeding on probing (BOP) (%)**		
T0	48.63 ± 12.74 ^a^	49.82 ± 14.5 ^a^
T3	20.60 ± 8.03 ^b^	11.90 ± 8.07 ^c^
T6	20.70 ± 9.44 ^b^	14.32 ± 9.07 ^c^
**Gingival recession (GR) (mm)**		
T0	0.84 ± 0.39 ^a^	0.81 ± 0.30 ^a^
T3	1.01 ± 0.60 ^a^	1.72 + 0.51 ^c^
T6	1.04 ± 0.63 ^a^	1.73 ± 0.54 ^c^
**Probing pocket depth (PPD) (mm)**		
T0	7.92 ± 0.93 ^a^	7.99 ± 0.83 ^a^
T3	5.31 ± 0.57 ^b^	4.11 ± 0.46 ^c^
T6	5.28 ± 0.67 ^b^	4.03 ± 0.49 ^c^
**Clinical attachment loss (mm)**		
T0	8.76 ± 1.32 ^a^	8.8 ± 1.13 ^a^
T3	6.32 ± 1.17 ^b^	5.83 ± 0.97 ^c^
T6	6.32 ± 1.3 ^b^	5.76 ± 1.03 ^c^

Identical letters indicate the absence of a statistically significant difference, while the difference in letters indicates a statistically significant difference. *p*-value < 0.0001. the values represent the mean values and standard deviations. T0 = before intervention; T3 = at 3 months of follow-up; T6 = at six months of follow-up.

## Data Availability

Upon reasonable request, the corresponding author M.E.M. can share any requested data.
